# Comparative evaluation of non-genetic factors affecting milk yield and composition of Red Dane and Jersey cattle in Zimbabwe

**DOI:** 10.1186/2193-1801-3-88

**Published:** 2014-02-14

**Authors:** Godfrey Bernard Nyamushamba, Tinyiko Edward Halimani, Venancio Edward Imbayarwo-Chikosi, Bruce Tavirimirwa

**Affiliations:** Faculty of Agriculture, Women’s University in Africa, P.O. Box, MP 1222, Mt Pleasant, Harare, Zimbabwe; Department of Animal Science, Faculty of Agriculture, University of Zimbabwe, P.O. Box, MP 167, Mount Pleasant, Harare, Zimbabwe; Department of Research and Specialist Services, Matopos Research Institute, P Bag k5137, Bulawayo, Zimbabwe

**Keywords:** Non-genetic factors, Month of calving, Calving interval, Age at calving

## Abstract

A study was carried out to evaluate non genetic factors affecting milk yield and milk composition in Zimbabwean Red Dane and Jersey cattle cattle. A total of 1004 and 10 986 unedited Red Dane and Jersey 305-day lactation records respectively, were obtained from Livestock Identification Trust (LIT) containing 22 herds (1 Red Dane herd and 21 Jersey herds), with Red Dane calving in the period 2004 to 2009 (giving year of birth from 1998 to 2007) and Jersey cows calving in the period 1996 to 2008 (giving year of birth from 1994 to 2005). The General Linear Model (GLM) procedure of the Statistical Analysis System (SAS, 2004) version 9.1.3 was used to determine the genetic parameters and environmental factors. Calving interval, month of calving, parity and quadratic effects of age at calving fitted as covariates significantly (*P* < 0.0001) affected the milk, fat and protein yields. Milk, fat and protein yields obtained increased with an increase in calving interval. There was a linear and quadratic relationship between the production traits and age at calving of the Jersey cattle implying that milk, fat and protein yields increase with age of the animal. It is thus important to preadjust data for these environmental factors when carrying out genetic evaluations of production traits in dairy cattle.

## Introduction

Non-genetic factors in animal production are those effects that are not part of the genetic make-up of an animal. These factors are not transmitted from parent to offspring (Nyamushamba et al., 2013). When the genetic effect on a trait is weak, it is lowly heritable and the environment has the greatest influence on that trait. Environmental factors tend to obscure the animal’s true genetic ability. (Missanjo et al. 2011) observed that selection within the best environment allowed better gene expression and selection response were therefore improved. Environmental variance, which by definition embraces all variation of non-genetic origin, is a source of error that reduces precision in genetic studies.

Milk, butterfat and protein yields are some of the factors that drive economic profitability of dairy farms. Therefore, striving to increase milk, butterfat and protein yields per animal, while decreasing feed and other costs, can lead to economic gains in farms. Whilst it is generally recommended that animals should be selected within the environment in which the animals and their progeny are reared, the magnitude of environmental influence should be considered (Nyamushamba et al. 2013). Efforts to improve traits that are greatly influenced by the environment should primarily focus on managerial inputs that modify the conditions under which the genotypes are expected to perform. The variation in milk, butterfat and protein yields can be attributed to several non genetic factors. These include age at calving, calving interval, days dry, season (month) of calving, herd and parity. Research has shown that in Zimbabwe, agro ecological regions affect milk production (Kunaka and Makuza, 2005; Missanjo, 2010).

The study aimed to establish environmental factors that affect milk, butterfat and protein yields for Jersey and Red Dane cattle in Zimbabwe. The information on environmental factors affecting milk production may reveal the need to have matings and hence calvings that occur at certain times of the year that gives higher yields with less input. It is therefore critical to provide information on both the genetic and non genetic factors that influence milk production. A study on the non-genetic-factors affecting milk production in Red Dane and Jersey cattle is therefore justifiable. The results can be used as a management tool, to improve selection criteria by accounting for non-genetic factors. The objective of this study was to determine the effect of non genetic factors on milk, protein and fat yields.

## Materials and methods

### Environment

Zimbabwe is located in Southern Africa in the tropical savannah region. The total land is 390,759 km^2^ and it is divided into five agro-ecological regions. Rainfall patterns and crop production progressively deteriorate from regions I to V. However, livestock production including dairying is practiced in all the regions. In the regions with low rainfall, dairying is assisted by production of drought-resistant fodder crops. Most dairy farms are located within 40 km of the major cities and towns (USDA, 2009).

### Data and data edits

The standard 305-day milk production records of pure bred Red Dane and Jersey were obtained from Zimbabwe Livestock Identification Trust. (Nyamushamba 2012) described the data set and the edits. This gave a data set of 10,986 and 1 321 records with Jersey and Red Dane cows calving in the period 1996–2008 and 2004–2009 respectively. Records were of individual cow milk yield, butterfat and protein contents. Parity of the lacting cows was from parity 1 to 7. Calving interval was recorded from 320 days to 455 days. Month of calving was from January to December. All the 12 months were considered since cows calved at different months of the year.

### Statistical analysis

The data were analysed using an animal model of the General Linear Model (GLM) of Henderson Type III sum of squares in Statistical Analysis Systems version 9.1.3 (SAS 2004). The data was fitted to the following animal model;

Where:

Y_ijk_ is the observed value for all milk traits (305-day milk yield; fat and protein yields);

μ is the overall mean common to all observation;

MOC_i_ is the fixed effect of herd-year-season _i_ (i = 1,2,3,…,12);

CI_j_ is the fixed effect of calving interval _j_ (j =1,2,3,…,10);

P_k_ is the fixed effect of parity k (k = 1,2,3,…,7)

b_1_ and b_2_ are the linear and quadratic regression coefficients respectively on age at calving (AC) in months; and

e_ijk_ is the random animal effect, e_ijk_ ~ (N (0,σ^2^_e_)

## Results and discussion

**Figure 1:**
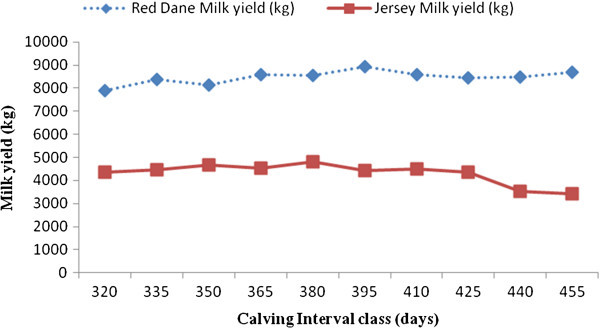
**Comparative effect of calving interval on milk yield for Red Dane and Jersey cattle.**

**Figure 2:**
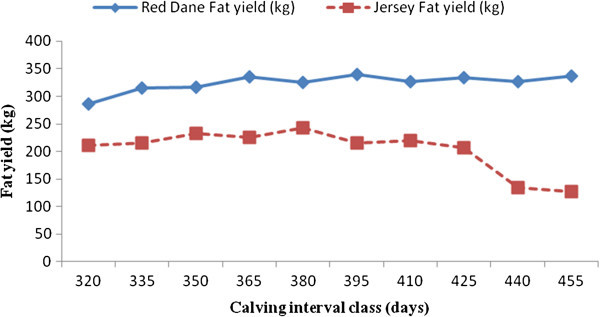
**Comparative effect of interval on fat yield for Red Dane and Jersey cattle.**

**Figure 3:**
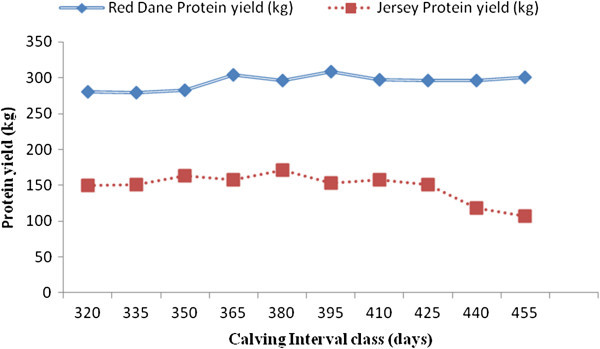
**Comparative effect of calving interval on protien yield for Red Dane and Jersey cattle.**

**Figure 4:**
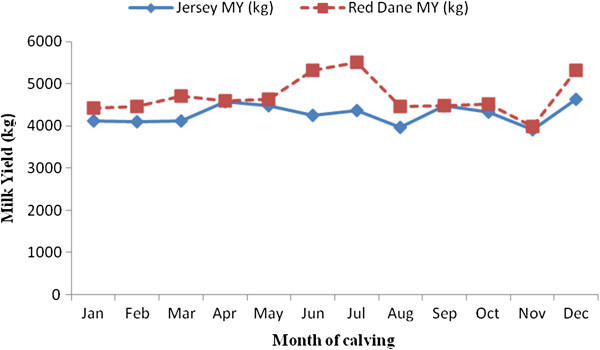
**Comparative effect of month calving on milk yield for Red Dane and Jersey cattle.**

**Figure 5:**
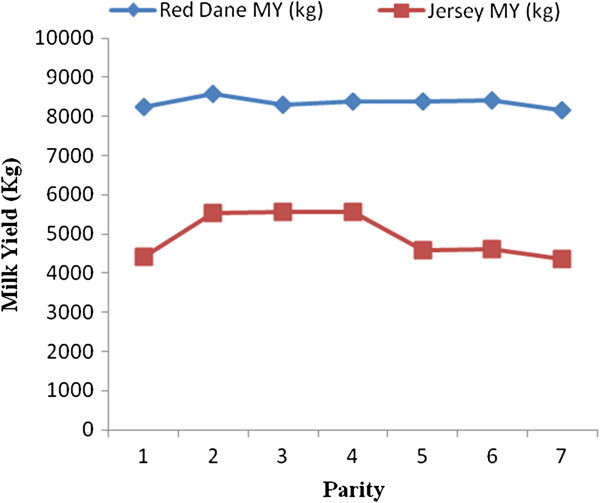
**Comparative effect of parity on milk yield for Red Dane and Jersey cattle.**

Factors which significantly affect milk, butterfat and protein yields in Zimbabwean Red Dane and Jersey cattle are shown in Figures 1, 2, 3, 4, and 5. Calving interval, parity and month of calving had a significant (P < 0.0001) effect on milk, fat and protein yields of Red Dane and Jersey cattle in Zimbabwe. The results found in this study are consistent with literature (Makuza 1995; Mandizha 1998; Imbayarwo-Chikosi 1999; Kunaka 1999; Amimo et al. 2007; Missanjo 2010; Nyamushamba et al. 2013). It is expected that the different herds have different levels of production because of variations in the level of management. For instance, in herds where disease control is high and feeding regime is also high, production is expected to be high.

### Comparative effect of calving interval on milk, butterfat and protein yields for Red Dane and Jersey cattle

Milk, butterfat and protein yields of the Red Dane and Jersey cattle were significantly affected by calving interval (P < 0.0001). These production traits demonstrated a continual increase in yield as the calving interval increased (Figures 1, 2 and 3). Figure 1 is illustrating that the Red Dane produces higher milk yield as compared to the Jersey cattle. The Red Dane cattle produced milk yield of an average of 8300 kgs whilst the Jersey cattle produced 4200 kgs. These results are in agreement with those reported by (Hatungumukana et al. 2007), (Missanjo et al. 2011) and (Nyamushamba et al. 2013) that calving interval significantly affect milk yield and composition of dairy cattle. The ideal calving interval is 365 days for both breeds in milk production whereby the producer is assured of one calf from each cow annually. This would also ensure that the cow can replenish its body reserves and regenerate secretory tissue without necessarily leading to over-conditioning.

### Comparative effect of month of calving on milk yield for Red Dane and Jersey cattle

Month of calving had a significant (P < 0.0001) effect on milk, butterfat and protein yields of Red Dane and Jersey dairy cattle in Zimbabwe. The months of June and July gave the highest milk yield for the Red Dane cattle. Milk yield for Jersey cattle was high in September and December. The results reported in this study are consistent with literature (Imbayarwo-Chikosi, 1999 and Missanjo, 2010) that herd-year-season significantly affect milk yield and composition of dairy cattle in Zimbabwe. It is expected that different herds have different levels of production because of variations in the level of management. Different month of calving and seasons in different agro-ecological zones can also contribute to the differences between herds (Kunaka, 1999). Differences in breeds also contribute differences in milk, protein and butterfat yields in Zimbabwe.

### Comparative effect of parity on milk yield for Red Dane and Jersey cattle

Parity had a significant effect (P < 0.05) on milk yield in Red Dane and Jersey cattle. High milk yield was recorded in parity 2 for the Red Dane cattle and there was a slight decrease in parity 3. There was a slight increase for parity 4, 5 and 6 and then a slight decrease in parity 7. The milk yield for the Jersey cattle increased gradually from parity 1 up to parity 4 and there was a decline in parity 5 with no significant difference in the milk yield in parity 1 and 6. In parity 7 there was a slight decrease. Milk yield was highest in parity 4 and the lowest in parity 5 for the Jersey cattle. The red Dane produced more milk yield compared to the Jersey cattle and this was illustrated in Figure 5 above.

There was a gradual increase in milk yield from parity 1 to 4 in Jersey cattle. This suggests that milk yield increases as parity proceeds because large cows produce more milk than small cows due to large body size and increased udder development that comes with repeated pregnancies as well as full development of tissues of the udder. Also, multiparous cows reach their peak earlier in the lactation than first parity cows and consume more feed, eat larger meals and drink more water therefore their persistency will be longer than that of the first calvers leading to less milk yield produced by first calvers. The milk yield was also lower in early parities because the feed that was provided to the heifers was also channeled to their growth as they were still growing. As the parities proceeded, milk yield increased because the feed requirements for growth were declining (Keown and Everett 1986).

Milk yield declined in parity 5 and 6, however there was no significant difference between the milk yield in parity 1, 5 and 6. The results were in line with literature from the study of Aysrhire and Holstein breed (Amimo et al. 2007). The decline is due to decline in body condition and degeneration of the body system over the recurring pregnancies. It will depend on whether the cow is able to maintain the condition to a subsist level. Therefore, it can be concluded that the productive period of Red Dane cows is as long as that of other predominant exotic breeds (Amimo et al. 2007). They can sustain the dairy enterprise for it is possible for them to provide the farm with acceptable levels of milk yield and composition from parity 1 up to parity 6 or 7, just as other exotic breeds. Also, the cows can be bred up to parity 7 before they are culled, being a desirable trait.

The results of this study were similar to findings in other studies made in the Tropics with Holstein and Ayrshire breeds (Amimo et al. 2007) where the peak was obtained in parity 4. The rate of culling in Red Dane and other exotic breeds, relative to parity, is comparable. This can be supported by results that were obtained by (Amimo et al. 2007) where the Holstein cows were culled in parity 6 when milk yield had dropped, leading to a sudden increase in parity 7. These results disclose that that the effect of parity on Red Dane and other predominant breeds in the large scale sector is comparable. Basing on the effect of parity, performance of Red Dane is similar to that of the other predominant breeds in the large sector therefore can also be adopted at a larger scale as the other exotic breeds

## Conclusion

Month of calving, parity and calving interval significantly affected milk, butterfat and protein yields for Red Dane and Jersey cattle in Zimbabwe. Milk, butterfat and protein yields increased with an increase in parity in both breeds. It is thus necessary to pre adjust data for these environmental factors when carrying out genetic evaluations of production traits in Zimbabwean Red Dane and Jersey cattle. The Red Dane gave the highest milk yield in June and July whilst in August to November its milk yield decreased. Jersey milk yield was high in the months of September and December. This is because the Jersey is heat tolerant during the hot season.
